# Tackling Conifer Needle Cast and Ash Dieback with Host-Derived Microbial Antagonists Exhibiting Plant Growth-Promoting Traits

**DOI:** 10.3390/microorganisms13112517

**Published:** 2025-10-31

**Authors:** Milana Šilanskienė, Dorotėja Vaitiekūnaitė, Vaida Sirgedaitė-Polikaitienė

**Affiliations:** Laboratory of Forest Plant Biotechnology, Institute of Forestry, Lithuanian Research Centre for Agriculture and Forestry, LT-53101 Kaunas, Lithuania; milana.silanskiene@lammc.lt (M.Š.); vaida.polikaitiene@lammc.lt (V.S.-P.)

**Keywords:** *Fraxinus excelsior*, *Hymenoscyphus fraxineus*, *Pinus sylvestris*, *Lophodermium seditiosum*, in vitro, bacteria, endophytes, epiphytes

## Abstract

Needle cast (*Lophodermium seditiosum* Minter, Staley & Millar) in Scots pine (*Pinus sylvestris* L.) and European ash (*Fraxinus excelsior* L.) dieback (*Hymenoscyphus fraxineus* (T. Kowalski) Baral, Queloz & Hosoya) are among the most destructive forest and tree plantation diseases in Europe, threatening not only targeted plant species but also the whole ecosystem. While considerable research effort has focused on microbial antagonists against ash dieback, comparable investigations into needle cast biocontrol remain virtually absent from the literature. Here, isolated microbial antagonists from European ash and Scots pine were evaluated for their efficacy against respective pathogens. In vitro dual-culture assays revealed bacteria with strong inhibitory effects on pathogen growth, as well as multiple plant growth-promoting traits (PGPTs). It was found that bacteria from the genera of *Pantoea*, *Erwinia*, *Priestia*, and *Pseudomonas* inhibited the growth of *H. fraxineus* by ≥70%. Most significantly, our investigation revealed that bacteria isolated from Scots pine, belonging to the genera *Pseudomonas*, *Bacillus*, and *Priestia*, inhibited the growth of *L. seditiosum* by 50% to 80%, representing one of the first reported bacterial antagonisms for this neglected pathogen. All isolates were positive for at least two PGPTs, primarily due to mineralization of organic phosphate and the production of siderophores. The dual functional traits of isolated bacteria highlight their potential application in integrated forest protection strategies, particularly for the previously overlooked *L. seditiosum* pathosystem.

## 1. Introduction

Scots pine (*Pinus sylvestris* L.) and European ash (*Fraxinus excelsior* L.) are common and highly valued tree species in Europe, providing essential ecosystem services such as timber production, carbon sequestration, and biodiversity support [1,2,3]. It was found that almost a thousand species of invertebrates, lichens, fungi, and bryophytes are associated with European ash, and out of those, 44 are obligate species that can only thrive symbiotically [4]. However, both tree species are facing significant threats from invasive and endemic pathogens. Needle cast disease, caused by the fungi *Lophodermium seditiosum* (Minter, Staley & Millar), severely reduces the growth and survival of younger Scots pine [5], while ash dieback, caused by *Hymenoscyphus fraxineus* ((T. Kowalski) Baral, Queloz & Hosoya), has devastated ash populations across the continent [6]. Forest diseases like these not only compromise forest productivity but also endanger genetic resources and long-term forest resilience.

Needle cast affects young (1–5 years) Scots pine trees, causing needle browning, drying, and premature fall, reducing growth by up to 48% in height and 17% in thickness [7]. Infection is strongly influenced by humidity and macroclimate, with outbreaks in dense, moist nurseries lasting up to 2–3 years [8,9].

European ash is affected by *Hymenoscyphus fraxineus*, which is responsible for the widespread ash dieback epidemic in Europe [10]. The pathogen, first reported in Poland about 30 years ago [11], originated in Asia, where it acts as a benign endophyte of *Fraxinus mandshurica* [12]. In Europe, however, *H. fraxineus* affects both young and mature trees, with only ~2% showing genetic resistance [13].

Both pathogens rely on efficient spore dispersal and tissue colonization, ultimately weakening host trees and increasing mortality risk [2,14]. Needle cast can be managed through silvicultural practices and fungicide sprays [15], no effective control exists for ash dieback [16]. Conventional measures are often limited and impractical in large-scale forests, while stricter fungicide regulations highlight the need for sustainable, eco-friendly disease management strategies [17].

The use of microbial biocontrol agents in both agriculture and silviculture has gained increased attention as effective tools in plant disease management. Antagonistic microorganisms can affect the pathogen in different ways: by producing harmful substances that inhibit the growth of the pathogen, by outcompeting for resources, by inducing the tree’s immune system, or by forming a protective physical barrier [18]. Antagonists may also possess plant growth-promoting traits (PGPTs) that can help the plant absorb nutrients more easily and better withstand biotic and abiotic stressors [19]. Such dual activity makes microbial agents particularly attractive for forest applications, where treatment should support both disease suppression and tree vigor in an eco-friendly and sustainable way.

Research was conducted on the engagement of plant application with microorganisms from the genus *Bacillus* on wheat [20], *Pseudomonas* on tomato and potato [21], and *Trichoderma* on mahogany and rain trees (*Samanea saman* (Jacq.) Merr.) [22] that demonstrated positive results in increased nutrient absorption and/or suppression of pathogens. *Heterobasidion* root rot, which occurs in Scots pine stands, can be managed with the fungus *Phlebiopsis gigantea* ((Fr.) Jülich), which quickly colonizes tree stumps and utilizes the nutrients needed for the pathogen (competition) [23]. Microbial antagonists, isolated from vineyards, were applied to grapevines infected with the fungal pathogen *Phaeomoniella* sp., resulting in a 38% reduction in necrosis [24], demonstrating successful biocontrol.

European ash and Scots pine native microorganisms offer distinct advantages for isolating biocontrol agents due to their established ecological relationships and adaptive compatibility with the host [25]. Native endophytes have co-evolved with their host-trees through extensive host–microbe interactions, resulting in sophisticated molecular dialogues and metabolic complementarity. This co-evolution enables superior colonization efficiency and compatibility with existing microbial communities compared to foreign species. Indigenous microorganisms minimize the risks of unintended ecological consequences, as non-native biocontrol agents may disrupt microbial equilibria or interact unpredictably with other species within the ecosystem [26]. However, systematic evaluation of host-derived microbial antagonists against ash dieback and needle cast, especially, remains limited, and this study addresses that gap by presenting one of the first comparative assessments of native bacterial isolates from Scots pine and European ash with antagonistic potential against both *L. seditiosum* and *H. fraxineus*, providing new insights into host-specific biocontrol interactions.

This study aimed to isolate and characterize bacteria from Scots pine and European ash and evaluate their antagonistic activity against *L. seditiosum* and *H. fraxineus*. Specifically, bacterial strains isolated from host tissues were evaluated for their antagonistic activity under in vitro conditions, and their plant growth-promoting properties were assayed. By integrating pathogen suppression and PGPTs, this study contributes to the development of sustainable, host-derived biocontrol strategies for forest health management.

## 2. Materials and Methods

### 2.1. Isolation and Identification of Bacteria

**Figure 1:**
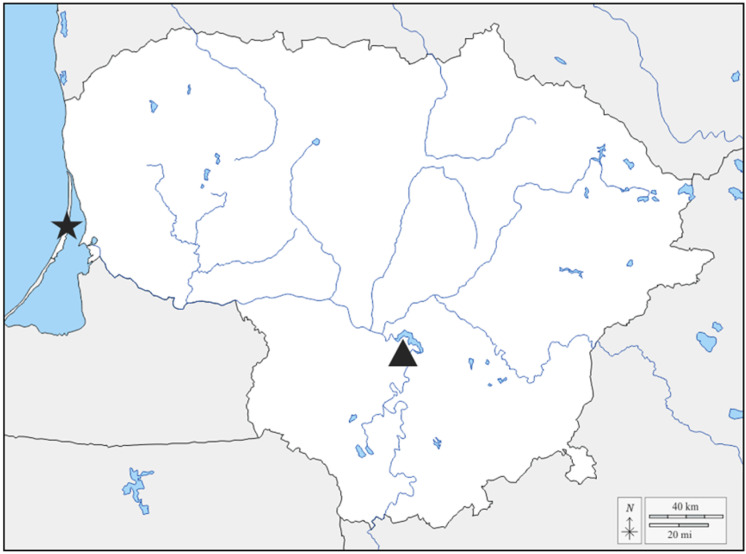
Map of Lithuania showing sample collection locations. The star-shaped icon represents the Scots pine sample collection location, and the triangle represents the European ash sample collection location.

Young ash twigs with leaves were collected in a European ash stand mixed with *Alnus glutinosa* L. and *Quercus robur* L. trees in Dubravai Forest, Kaunas distr., Lithuania (54.828370, 24.101225) in late May 2024 (Figure 1). Selected 30 European ash trees varied in age, but all were mildly suffering from ash dieback. Samples were gathered, put into sterile plastic bags, placed on ice, and immediately transferred to a refrigerator (4 °C) [27]. Prior to the next step, the samples were stored for approximately 20 h. The next day, ash leaves were surface sterilized under sterile conditions in a laminar flow hood as follows: leaves were soaked in sterile distilled water (dH_2_O) and then transferred to 70% ethanol (Stumbras, Kaunas, Lithuania) for 1 min. They were subsequently washed three times with sterile dH_2_O for 30 s each time. Afterward, leaves were soaked in 2% commercial bleach solution for 3 min, then washed 3× with sterile dH_2_O for 30 s each time.

Whole twigs with needles and buds from Scots pine were collected in a Scots pine stand located in Kuršių nerija, Juodkrantė Forest, Klaipėda district, Lithuania (55.533799, 21.10719) in late May 2024 (Figure 1). Thirty trees of varying ages that appeared relatively healthy were selected. The collected samples were immediately put into sterile plastic bags and placed on ice, and later stored in a refrigerator at 4 °C. The next day, the needles were sterilized under sterile conditions in a laminar flow hood. First, the needles were soaked in a solution for 5 min (125 mL sterile dH_2_O with a few drops of Tween 80 (Carl Roth, Karlsruhe, Germany)) and then washed three times for 2 min with sterile dH_2_O. Next, the needles were transferred to a 75% ethanol (Stumbras, Kaunas, Lithuania) solution for 30 s and then rewashed three times with sterile dH_2_O. Finally, the needles were soaked in a 2% commercial bleach solution for 5 min and washed three times with sterile dH_2_O for 30 s each time.

After sterilizing plant material, ash leaves were ready to be cut into small sections. The pine needles and buds were ground with a pestle and mortar to be placed on the growth medium (LB agar, modified M9 minimal salts medium, Water Yeast Extract medium) and incubated for a month in a growth chamber (BINDER BD 115, BINDER GmbH, Tuttlingen, Germany) at 27 °C in the dark.

To ensure a greater variety of isolated microbes, 3 different types of growth medium with varying nutrient levels were used [28]. LB agar was used because it is a rich medium suitable for fast-growing, generalist bacteria (10 g L^−1^ tryptone, 5 g L^−1^ yeast extract, 5 g L^−1^ NaCl, 15 g L^−1^ agar, pH 7 (Condolab, Madrid, Spain)). Modified M9 minimal salts medium was chosen as it favors slow-growing, metabolically independent endophytic bacteria (10 g L^−1^ Na_2_HPO_4_, 3 g L^−1^ KH_2_PO_4_, 0.6 g L^−1^ NaCl, 10 g L^−1^ (NH_4_)_2_SO_4_, 5 g L^−1^ glucose, 20 g L^−1^ agar, pH 7.2) and Water Yeast Extract Agar (WYEA), a low-nutrient medium, was included to support slow or stress-adapted plant-associated bacteria (0.25 g L^−1^ yeast extract, 0.5 g L^−1^ K_2_HPO_4_, 18 g L^−1^ agar, pH 7.2). pH was regulated with either NaOH (Merck, Darmstadt, Germany) or HCl (Tarchem, Tarnowskie Góry, Poland) solutions.

It was essential to inspect whether plant material sterilization was successful. To check, the final wash water was transferred to Malt Medium (MM) (22 g L^−1^ maltose, 8 g L^−1^ yeast extract, 6 g L^−1^ peptone, 20 g L^−1^ glucose, 15 g L^−1^ gelrite, pH 5.5) to assess if there were any non-endophytic fungi, and LB agar medium, to examine for possible bacterial contamination [29]. Petri dishes were monitored for a month to check for the growth of microorganisms. In the absence of growth on the plates, it was concluded that plant material disinfection was successful and that the emerged bacteria were, in fact, endophytic.

Independently, 3 epiphytic bacteria associated with European ash were isolated. Immediately after gathering plant material, leaves from 15 trees were placed on previously used LB, M9, and WYEA media to isolate easily cultivated epiphytes for use in further experiments.

Bacterial samples were further recultivated multiple times using the quadrant streaking method and purified.

For genetic identification, the selected purified bacterial cultures were sent to a sequencing center for DNA extraction and *16S rRNA* gene fragment sequencing (Macrogen Europe BV, Amsterdam, The Netherlands). Universal primer set 785F (GGATTAGATACCCTGGTA) and 907R (CCGTCAATTCMTTTRAGTTT) were used. Using Chromas version 2.6.6 (Technelysium Pty Ltd., South Brisbane, Australia) software, reverse primer sequences were reversed to forward complement sequences. Then, sequences were further edited with BioEdit software, version 7.7 (Informer Technologies, Inc., Los Angeles, CA, USA) and then aligned using standard parameters in NCBI (National Center for Biotechnology Information’s) BLASTn alignment tool, version 2.15.0. Later, fragments were matched with the NCBI database (BLAST Targeted Loci Nucleotide System) for Bacteria and Archaea (megablast) using ≥99% identity and ≥99% query coverage.

### 2.2. Dual Culture Antagonism Assay

Selected isolated bacterial colonies were tested for their potential to grow on a solid MM with added ash leaves before the experiment to see if bacteria can grow on this type of medium. Fresh colonies were streaked on said medium, and bacterial growth was observed for one week. Bacteria that could not proliferate on MM were dismissed from further tests.

For this experiment, *H. fraxineus* was obtained from the Westerdijk Fungal Biodiversity Institute, strain number CBS 122191, which originated from Austria. *L. seditiosum* was obtained from Leibniz Institute DSMZ-German Collection of Microorganisms and Cell Cultures (DSM 5029). Plugs of young, actively growing mycelium (8 mm diameter) were transferred to freshly prepared MM (with added ash leaves for experiments with *H. fraxineus*) to the center of the Petri dish [30], and mycelium radial growth was measured for 4 weeks. Twelve bacteria were tested against the pathogenic *H. fraxineus* (Hf), and three bacteria against the pathogenic *L. seditiosum* (Ls). Antagonism screening was performed in triplicate using fresh bacterial colonies and actively growing pathogen mycelium each time.

For each antagonism assay, fresh MM (for Hf, the medium was supplemented with 50 g L^−1^ frozen European ash leaves) was prepared in Ø90 mm plastic Petri dishes. Briefly, four bacterial smears were streaked on the medium in the shape of a square with a pathogen (Hf/Ls) mycelium plug in the center of the plate. One replicate consists of one bacterium and one pathogen plug (8 mm diameter) per plate. A plate with only the pathogen was used as a Control (untreated with bacteria). The experiment was considered finished when the pathogens’ mycelium reached the edges of the Control plate. Measurements of pathogen radius were taken at three locations over a 3-week period (starting from week 2) to check for any changes in possible antagonistic activity during this time. Pathogen growth inhibition was calculated as follows:

(1) $$I=\left.\left(\frac{C-T}{C}\right.\right)\times 100$$

where *I*—growth inhibition, *C*—average radius of the pathogen (Hf/Ls) in Control plates, *T*—average radius of the pathogen (Hf/Ls) in plates with bacteria.

### 2.3. Plant Growth-Promoting Traits (PGPTs)

Screening for PGPTs was completed in triplicate using fresh bacterial colonies each time. Isolated bacteria were tested for their potential to solubilize and mineralize phosphates, solubilize potassium, fix nitrogen, produce siderophores (iron-scavenging molecules), and indole-3-acetic acid (IAA, a growth-promoting hormone), and form biofilms (can help with bacterial colonization). These assays were selected because they represent key plant growth-promoting traits that collectively enhance host nutrition and vigor while contributing to pathogen suppression through improved nutrient acquisition, hormonal modulation [31]. Bacteria were screened using selective media: the prepared medium was poured into Petri dishes, and bacterial colonies were inoculated and incubated for a week. The exact methods are briefly described here.

Bacterial isolates were tested for phosphate solubilization and mineralization. Briefly, there were two types of prepared medium used: the first one with tricalcium phosphate as a P source (testing the ability to solubilize inorganic phosphate) and the second one with soy lecithin (testing the ability to mineralize organic phosphate) [32]. After incubation, plates were examined to determine if clear zones around inoculated bacteria were present. This indicated that the isolates were capable of phosphate solubilization/mineralization.

To evaluate K solubilization, Aleksandrow’s agar (Himedia, Mumbai, India) was used [33]. Clear zones around the colonies indicated that isolates were able to solubilize potassium. N fixation was tested by using a nitrogen-free Jensen’s medium (Himedia, India) [34]. Bacteria were considered diazotrophic if colony growth had well-defined growth zones.

The ability to produce siderophores was tested by using a modified Chromeazurol S (CAS) assay [35]. Fresh CAS reagent was prepared [36]: 0.182 g CTAB was solubilized in 100 mL of ddH_2_O and heated (1); 0.027 g FeCl_3_ × 6H_2_O was dissolved in 100 mL HCl (10 mM) (2); 0.06 g chromeazurol S dissolved in 50 mL of ddH_2_O (3). Then, 10 mL of FeCl_3_ × 6H_2_O solution (2) is poured into CAS solution (3) while constantly mixing, then adding 40 mL of CTAB solution (1) until the color changes to deep blue. Reagents were sterilized by autoclaving and then mixed 1:9 with LB medium, at pH 7.2 [35]. Media color changes from blue to orange/yellow were indicative of siderophore production.

To evaluate if bacterial isolates could produce phytohormone IAA (tryptophan-dependent), tests with Salkowski reagent were carried out. For this, bacteria were grown overnight in LB (Duchefa Biochemie, Haarlem, The Netherlands) with added tryptophan (0.15% *w*/*v*) in a thermal shaker (90 rpm, 30 °C), then 1.5 mL of this suspension was centrifuged, and 150 µL of the supernatant was transferred to microplates. The same amount of prepared Salkowski reagent was added (1 mL of 0.5M FeCl_3_ mixed with 49 mL of 35% HClO_4_ *v*/*v*), and the microplate was incubated in the dark for 30 min. The optical density (OD) was measured at 530 nm with a spectrophotometer (Synergy HT Multi-Mode Microplate Reader, Biotek Instruments Inc., Bad Friedrichshall, Germany), and the amount of produced IAA was calculated using a standard curve [35].

Bacteria were also checked for their ability to form biofilms. For this purpose, the modified tissue culture method was used [35]. Briefly, bacteria were grown in LB overnight, and then 2 µL of the suspension was transferred into a sterile 96-well microplate. Next, 198 µL of LB with added 1% glucose was incubated overnight. The next day, the microplate was washed 3× with sterile dH_2_O and left to air-dry. After drying, the biofilm layer was dyed using a 0.1% Gentian violet solution for 10 min, and the microplate was washed three times. Then, the biofilm layer was solubilized using 300 µL 96% ethanol for 30 min, and OD was measured with a spectrophotometer (Synergy HT Multi-Mode Microplate Reader, Biotek Instruments Inc., Bad Friedrichshall, Germany) at 630 nm with 96% ethanol as a control. Finally, the relative biofilm strength was calculated as follows: ODc = average OD of control + 3× the standard deviation of control. Weak biofilm is considered around the same OD as control, moderate—around 2–4 ODc, and strong biofilm was considered 4 < ODc [37].

### 2.4. Statistical Analysis

Results were compiled and visualized using Microsoft Office Excel, version 2406. Statistical analyses were conducted using SPSS, version 28.0.1.1 (IBM Inc., Armonk, NY, USA), employing the Kruskal–Wallis H test (*p* < 0.05) and post hoc pairwise comparison using Dunn’s test. 

## 3. Results

### 3.1. Antagonistic Activity in Dual Culture Assay 

**Figure 2:**
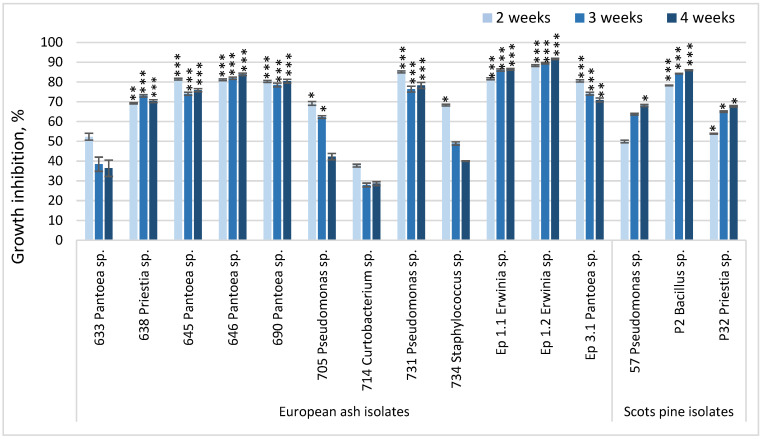
Antagonistic activity of isolated endophytic and epiphytic bacteria from European ash against ash dieback phytopathogen *H. fraxineus* and bacteria from Scots pine against needle cast phytopathogen *L. seditiosum*. Measurements of Hf/Ls radius were taken until week 4. The graphs use relative standard error (RSE) bars. Statistically significant differences from the control (Hf/Ls with no bacteria) group were determined by Kruskal–Wallis H test: * *p* < 0.05; ** *p* < 0.01; *** *p* < 0.001.

Inhibition of *Hymenoscyphus fraxineus* (Hf) and *Lophodermium seditiosum* (Ls) mycelial radial growth was observed over four weeks, with fungi radius measurements taken at weeks 2, 3, and 4 (Figure 2). The Kruskal–Wallis H test confirmed statistically significant differences in pathogen growth among treatments across the second, third, and fourth weeks of measurement. For both the Hf and Ls assays, significant effects of bacterial inoculation were observed at week 2 (H = 100.709, *p* < 0.0001), week 3 (H = 100.007, *p* < 0.0001), and week 4 (H = 100.746, *p* < 0.0001), indicating progressive inhibition of pathogen colony expansion by several isolates compared with the uninoculated control. Pairwise comparisons revealed that both endophytic and epiphytic isolates markedly reduced pathogen colony radius, with the strongest inhibition usually recorded at week 4. These findings align with the graphical representation in Figure 2.

**Figure 3:**
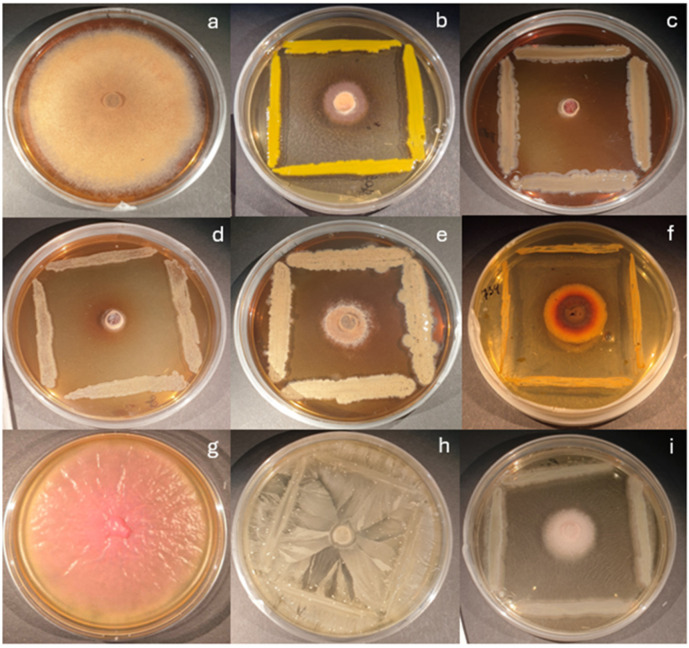
Dual-culture antagonism assay after 3 weeks. Against *H. fraxineus*: (**a**)—control (Hf), (**b**)—705 *Pseudomonas* sp., (**c**)—Ep 1-1 *Erwinia* sp., (**d**)—Ep 3-1 *Pantoea* sp., (**e**)—638 *Priestia* sp., (**f**)—734 *Staphylococcus* sp.; against *L. seditiosum*: (**g**)—control (Ls), (**h**)—P2 *Bacillus* sp., (**i**)—P32 *Priestia* sp.

The most promising endophytic bacterial isolates were 638—*Priestia* sp., 645, 646, 690—*Pantoea* spp., and 731—*Pseudomonas* sp., as well as epiphytes Ep1-1 and Ep1-2 belonging to the genus of *Erwinia*, and Ep3-1 from the *Pantoea* genus, that managed to inhibit the growth of Hf by ≥70% for all four experimental weeks (Figure 2 and Figure 3). Bacteria from *Curtobacterium* and *Staphylococcus* were less effective, although they did inhibit the growth of the pathogen by at least ~30%. As for Scots pine, the most promising bacterial isolates were P2, belonging to the *Bacillus* genera, which inhibited the growth of Ls by 80%, and P32, from the *Priestia* genera, which inhibited the growth of Ls by 50% in the first and 60% or more in the second and third experimental weeks.

### 3.2. Bacterial Identification

Sequencing of the *16S rRNA* gene fragment was used for the identification of the isolated bacteria. Identifications with >97% sequence identity were deemed preliminarily reliable at the species level, while those at 90–96% were assigned only a genus. In most cases, fragment lengths varied from 1457 to 1488 base pairs, and query coverage was 100%. Identification was assigned based on the highest sequence similarity match, and the results were summarized in Table 1. The genus *Pantoea* was the most prevalent among all identified European ash isolates, comprising five bacteria (633, 645, 646, 690, Ep 3-1). Sequencing results revealed that bacterial isolates 633, 645, and Ep 3-1 were highly similar to *Pantoea agglomerans*, with sequence identities exceeding 98% (fragment lengths of 1467, 1470, and 1457 bp, respectively). While isolate 646 was revealed to be *Pantoea alii* with 98.98% sequence identity (1467 bp), isolate 690 showed 99.17% identity match with *Pantoea vagans*, although the fragment length was only 625 bp. Sequence comparison of epiphytes Ep 1-1 and Ep 1-2 both indicated a 99.73% match with *Erwinia rhapontici*. Two bacterial isolates belonging to the genus *Pseudomonas* were examined: isolate 705 had a 99.66% identity match with *Pseudomonas baltica* (1466 bp), and isolate 731 had a 99.86% identity match with *Pseudomonas libanensis* (1467 bp). Bacterial isolate 638 was proven to be *Priestia aryabhattai* with a 100.00% identity match (1488 bp), isolate 714 demonstrated 100.00% identity match with *Curtobacterium flaccumfaciens* (1021 bp), and isolate 734 showed 99.93% identity match with *Staphylococcus haemolyticus* (1464 bp). As for Scots pine, bacterial isolate 57 was proven to be *Pseudomonas lutea* (1459 bp), isolate P2—*Bacillus velezensis* (1468 bp), and isolate P32 was proven to be *Priestia megaterium* with a 100.00% identity match (1462 bp).

Overall, almost all isolates could be reliably assigned to the species level (>97% sequence identity), while isolate 690 should be repeatedly sequenced because the final assembled sequence for it was only 625 bp long.

### 3.3. Plant Growth-Promoting Traits in Isolated Bacteria

**Figure 4:**
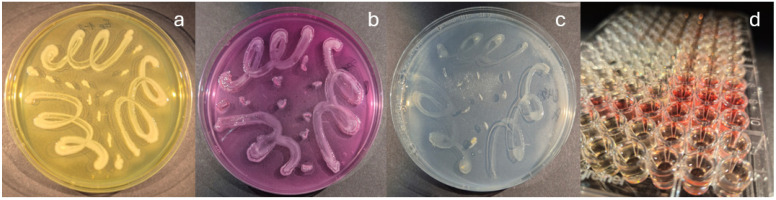
Plant growth-promoting traits: (**a**) siderophore production (medium color change from blue to yellow) and (**b**) organic phosphate mineralization of Ep 1-2 (*Erwinia* sp.), (**c**) solubilization of potassium (clear zones around the bacterial colonies) (**d**) and production of IAA (color change from yellow to red) by 645 (*Pantoea* sp.).

All tested isolated bacteria displayed PGPTs: at least three bacteria from European ash and at least one from Scots pine, as determined by the analyses (Table 2). Ten out of fifteen tested isolates were found to be capable of mineralizing organic phosphate. Interestingly, Ep 1-1 and Ep 1-2, belonging to *Erwinia* sp., induced unexpected medium color change from clear, transparent into bright pink (Figure 4b), which might be due to the species’ ability to produce water-soluble pink pigment in some media [38]. Unfortunately, none of the 15 tested bacteria solubilized inorganic phosphate. Only six isolates belonging to the genus *Pantoea* spp. (isolates 633, 645, 646) and *Pseudomonas* spp. (isolates 690, 731, and 57) solubilized potassium. Nine out of 15 bacteria had the potential to fix nitrogen, and eleven produced siderophores. The level of produced IAA varied after 3 days of incubation: ranging from 3.28 µg/mL (isolate 734, belonging to Staphylococcus) to 44.79 µg/mL (isolate 646, from the genus *Pantoea*). Most of the tested bacteria could form weak biofilms, except for isolates 633, 645, and Ep 3-1 from the genus *Pantoea* and isolate 731 from the genus *Pseudomonas*, which managed to form moderate-strength biofilms. Representative examples of PGPTs are displayed in Figure 4.

## 4. Discussion

Infection by *Lophodermium* needle cast in Scots pine begins in the above-ground parts of the tree, similarly to infection of Hf. Hence, among the first line of defense against the disease could be the hosts’ microbiota, offering a necessary eco-friendly solution adapted to the ecological niche of the host trees. Understanding the composition and functional potential of these microbial communities in different tree species is therefore essential for developing sustainable strategies against both Hf and Ls.

Endophytic and epiphytic microorganisms serve complementary biocontrol roles in forest trees: endophytes colonize internal tissues, forming stable associations that enable hormonal modulation, metabolite exchange, and systemic resistance against pathogens by accessing xylem and phloem to intercept invasion and strengthen internal defenses. Epiphytes colonize external surfaces, providing fast-acting protection through competitive exclusion, antimicrobial compounds, siderophores, and hydrolytic enzymes that occupy infection sites, limit spore germination, and enhance nutrient availability. For perennial hosts like ash and pine, integrating both epiphytes’ immediate surface antagonism with endophytes’ long-term systemic resistance offers the most effective strategy for sustainable disease suppression [39].

There have been more studies focused on ash fungal composition than on ash microbial composition, but this trend is slowly changing. The ash-associated microbial community is diverse and may depend on the tree’s condition. For example, studies have shown that the more tolerant European ash tree microbiome at the genus level consists of bacteria such as *Luteimonas*, *Aureimonas*, *Pseudomonas*, *Paenibacillus*, and *Bacillus*. These bacteria possess antifungal traits, including PGPTs, and may inhibit spore germination or produce extracellular enzymes to break down the fungal cell wall [40]. In previous studies, more tolerant European ash trees have been found to harbor fungi such as *Papiliotrema flavescens* Summerb.), which tested positive as potential biocontrol agents against Hf [41]. It was reported that fungal diversity on ash trees depends on the tissue type tested and the season, with more susceptible trees bearing a greater number of pathogenic fungi [42]. Building on this background, the current study’s initial step in curing ash dieback or limiting its impact was the isolation of easily cultivable bacteria from the phyllosphere of European ash. Twelve bacteria were isolated (genera *Pantoea*, *Erwinia*, *Pseudomonas*, and *Priestia*), and the findings are consistent with previous studies: there are reported cases of bacteria from the genera *Pantoea vagans* (Brady et al.), *Pseudomonas caspiana* (Busquets et al.), *Erwinia billingiae* (Mergaert et al.), *Priestia aryabhattai* ((Shivaji et al.) Gupta et al.) (formerly known as *Bacillus aryabhattai*) acting as antagonists (suppressing the ash pathogen up to 55%) [30]. In the current study, suppression of the pathogen growth may have been through antibiotic production or competitive exclusion; however, the precise mechanism is unknown. For example, isolate 731 (preliminarily identified as *Pseudomonas libanensis*, Dabboussi et al.) may employ antifungal secondary metabolites such as phenazines [43], while *Pantoea* spp. could compete for iron through siderophores, as indicated by our positive test for siderophore production [44].

Further supporting evidence for bacterial biocontrol potential comes from studies on *Priestia aryabhattai*. This bacterium possesses PGPTs, including phosphate solubilization, atmospheric nitrogen fixation, and the production of IAA [45] (as demonstrated in our investigation as well), as well as potassium and zinc solubilization. It has been described as multi-stress tolerant [46]. *P. aryabhattai* also showed antagonistic activity under in vitro conditions against potato bacterial wilt (caused by *Ralstonia solanacearum*, (Smith) Yabuuchi et al.), producing a 3.7 cm inhibition zone, while in planta treatment reduced disease incidence by 40% [45]. Similar antagonistic activity was reported against *Penicillium expansum* (Link) (via volatile 2-nonanol production) [47], *Magnaporthe oryzae* ((Catt.) B.C. Couch) (pathogen growth reduction of 62.31%)) [48], *Fusarium oxysporum* (Schltdl.) (by 85%), *Alternaria alternata* ((Fr.) Keissl.) (by 76%), *Rhizoctonia solani* (J.G. Kühn) (by 38%), and *Ustilaginoidea virens* ((Cooke) Takah.) (by 32%) [46]. Moreover, *P. aryabhattai* can produce extracellular enzymes, such as protease, cellulase, and lipase, which could potentially degrade the cell walls of pathogenic fungi [46]. Its reported nematicidal activity against *Heterodera glycines* (soybean cyst nematode)—causing egg hatch suppression and juvenile mortality [49]—further highlights its versatility and biocontrol potential. Collectively, these findings position *P. aryabhattai* as a promising candidate for future studies on ash dieback mitigation.

In parallel, the microbiota of Scots pine (*Pinus sylvestris*) also plays a vital role in tree health and disease resistance. Several studies have highlighted the ecological importance and diversity of the microbiota associated with Scots pine. Research has shown that *Bacillus* spp. and *Pseudomonas* spp. have been previously found in Scots pine tissues [50,51]; this pattern was also observed in the current study. Specifically, *Bacillus velezensis* (isolate P2), *Priestia megaterium* (isolate P32), and *Pseudomonas lutea* (isolate 57) were detected in Scots pine needles and buds. Other bacteria found in Scots pine tissues include genera such as *Variovorax*, *Novosphingobium*, *Sphingomonas*, *Microbacterium*, and *Methylobacterium* [50,52]. Previous research has demonstrated that the diversity of endophytic bacteria in conifers varies by type and season [51,53].

Among the isolates tested against Ls in vitro, three species originating from Scots pine (*Pseudomonas lutea* Peix et al., *Priestia megaterium* (de Bary) Gupta et al., and *Bacillus velezensis* Ruiz-Garcia et al.) showed promising antagonistic effects. *B. velezensis*, in particular, has proven effective against other fungal tree pathogens, such as *Bursaphelenchus xylophilus* (pine wilt disease causal agent) [54], *Fusarium oxysporum* (causing damping-off) [55], and *Sphaerulina musiva* (affecting *Populus*) [56]. These findings indicate that pine-associated bacteria may also serve as valuable biocontrol agents against *Lophodermium* species.

Beyond antifungal activity, several isolates demonstrated plant growth-promoting traits (PGPTs). Our findings reinforce previous studies reporting on *Pantoea* spp. and their PGPTs, including exopolysaccharides biosynthesis, ACC deaminase (1-aminocyclopropane-1-carboxylic acid), and the production of phytohormones (cytokinins, auxins, abscisic, and gibberellic acids) [44]. Similarly, *Pseudomonas* spp. are known for solubilizing zinc, expressing ACC deaminase activity, and producing ammonia [57]. Regarding *Erwinia* spp., our results showed that these isolates mineralized organic phosphate, fixed nitrogen, and produced siderophores for enhanced iron uptake. Previous research on *E*. *rhapontici* has demonstrated its nitrogen-fixing and phosphate-solubilizing abilities, a high carbon source utilization rate (70.52%) [58], and a positive impact on tree growth and secondary metabolite synthesis [59], although siderophore production was not detected in that study. In contrast, the *B. velezensis* isolate produced siderophores, while the literature indicates that it can also form biofilms [60], fix nitrogen, and solubilize both organic and inorganic phosphates [61]. Likewise, isolate 57 (*P. lutea*) exhibited potassium solubilization and siderophore production; previous studies further report its phosphate solubilization and auxin synthesis abilities [62].

Finally, it is worth noting that several bacteria are already applied in arboriculture to improve nutrient uptake, enhance plant growth, and protect trees against phytopathogens. *B. subtilis* has been tested against pine wilt disease [63] and cherry leaf spot [64] due to its ability to produce compounds that induce systemic resistance (ISR), promote plant growth via phytohormone secretion, reduce pathogen virulence, and enhance stress tolerance [65]. Similarly, *B. velezensis* produces antimicrobial compounds and volatile metabolites (iturin, fengycin, surfactin) that suppress pathogen growth and stimulate ISR under both biotic and abiotic stress [60,66]. *Pseudomonas syringae* van Hall, 1902, has been employed to control brown rot in peaches [67], enhancing nutrient uptake and producing phenazine, a potent antifungal and antibacterial metabolite [65]. Moreover, *Pantoea agglomerans* has been successfully applied to apple flower stigmas against fire blight (*Erwinia amylovora* (Burrill) Winslow et al.) through antibiosis [68]. Collectively, these examples highlight the remarkable biocontrol and plant growth-promoting potential of bacterial taxa identified within the ash and pine microbiota, paving the way for further application-oriented research.

## 5. Conclusions

In conclusion, native bacterial isolates from European ash and Scots pine exhibited promising biocontrol and plant growth-promoting potential. *Pantoea* (initially identified as *P. agglomerans*, *P. allii*, *P. vagans*), *Pseudomonas* (*P. libanensis*), *Erwinia* (*E. rhapontici*), and *Priestia* (*P. aryabhattai*) isolates from ash suppressed the ash dieback pathogen by ≥70% in vitro and showed multiple PGPTs, including organic phosphate mineralization, siderophore production, nitrogen fixation, and potassium solubilization. The *Bacillus* (*Bacillus velezensis*) isolate from Scots pine inhibited *Lophodermium seditiosum* growth by 80%, though it tested positive only for siderophore production. These findings represent a significant step toward the eco-friendly management of ash dieback and needle cast diseases. Further in planta and field studies are needed to confirm these effects and clarify the mechanisms of pathogen inhibition.

## Figures and Tables

**Table 1:** Results of sequence analysis of the *16S rRNA* gene fragment of the 12 bacteria isolated from *F. excelsior* phyllosphere (endophytes and epiphytes) and 3 bacteria isolated from Scots pine needles and buds used in this study.

	Bacteria Number	Closest NCBI Match	Accession No. *	Fragment Length, bp	% Identity	Query Coverage, %
European ash antagonistic bacteria	633	*Pantoea agglomerans*, NCTC9381	NR_114735.1	1467	98.98	100
	638	*Priestia aryabhattai*, B8W22	NR_115953.1	1488	100.00	100
	645	*Pantoea agglomerans*, NCTC9381	NR_114735.1	1470	98.98	100
	646	*Pantoea allii*, BD 390	NR_115258.1	1467	98.98	100
	690	*Pantoea vagans*, LMG 24199	NR_116115.1	625	97.76	100
	705	*Pseudomonas baltica*, MBT-2	NR_181571.1	1466	99.66	100
	714	*Curtobacterium flaccumfaciens*, BCCM/LMG 3645	NR_025467.1	1021	100.00	100
	731	*Pseudomonas libanensis*, CIP 105460	NR_024901.1	1467	99.86	100
	734	*Staphylococcus haemolyticus*, JCM 2416	NR_113345.1	1464	99.93	100
	Ep 1-1	*Erwinia rhapontici*, DSM 4484	NR_041976.1	1468	99.73	100
	Ep 1-2	*Erwinia rhapontici*, DSM 4484	NR_041976.1	1466	99.73	100
	Ep 3-1	*Pantoea agglomerans*, DSM 3493	NR_041978.1	1457	99.17	100
Scots pine antagonistic bacteria	57	*Pseudomonas lutea*, OK2 16S	NR_029103.1	1459	98.93	100
	P2	*Bacillus velezensis*, FZB42	NR_075005.2	1468	99.93	100
	P32	*Priestia megaterium*, ATCC 14581	NR_112636.1	1462	100.00	100

* Accession number in the NCBI Reference Sequence Database (https://www.ncbi.nlm.nih.gov/refseq; accessed on 16 February 2025).

**Table 2:** Plant growth-promoting trait assay results of the 12 bacterial isolates from European ash and the 3 bacterial isolates from Scots pine used in this study.

	Bacteria Number	Solubilization of Potassium	Solubilization of Inorganic Phosphate	Mineralization of Organic Phosphate	Nitrogen Fixation	Production of Siderophore	IAA Production, µg/mL	Formation of Biofilms
European ash antagonistic bacteria	633	+ *	−	+	−	+	12.87	moderate
	638	−	−	+	+	−	6.76	weak
	645	+	−	+	+	+	28.61	moderate
	646	+	−	+	+	+	44.79	weak
	690	+	−	+	+	+	20.19	weak
	705	−	−	+	−	+	9.12	weak
	714	−	−	−	+	−	7.96	weak
	731	+	−	+	+	+	7.79	moderate
	734	−	−	−	−	−	3.28	weak
	Ep 1-1	−	−	+	+	+	5.91	weak
	Ep 1-2	−	−	+	+	+	6.17	weak
	Ep 3-1	−	−	−	−	+	19.94	moderate
Scots pine antagonistic bacteria	57	+	−	−	−	+	5.49	weak
	P2	−	−	−	−	+	6.45	weak
	P32	−	−	+	+	−	5.56	weak

* Plus (+) indicates a positive reaction, and minus (−) indicates a negative response.

## Data Availability

The datasets presented in this article are not readily available because the data are part of an ongoing study. Requests to access the datasets should be directed to corresponding author.
